# The Clinical Relevance of Mast Cell Activation in Myalgic Encephalomyelitis/Chronic Fatigue Syndrome

**DOI:** 10.3390/diagnostics15222828

**Published:** 2025-11-07

**Authors:** Johanna Rohrhofer, Lilian Ebner, Johannes Schweighardt, Michael Stingl, Eva Untersmayr

**Affiliations:** 1 Institute of Pathophysiology and Allergy Research, Center for Pathophysiology, Infectiology and Immunology, Medical University of Vienna, 1090 Vienna, Austria; johanna.rohrhofer@meduniwien.ac.at (J.R.); lebner1@mgh.harvard.edu (L.E.); n51805061@students.meduniwien.ac.at (J.S.); 2 CerePrax, 1150 Vienna, Austria; ordination@neurostingl.at

**Keywords:** chronic fatigue syndrome/myalgic encephalomyelitis (CFS/ME), post-infection syndromes, mast cell activation, immunology, chronic inflammation, autonomic nervous system, patient stratification

## Abstract

**Background/Objectives:** Growing evidence suggests that mast cell activation (MCA) may contribute to Myalgic Encephalomyelitis/Chronic Fatigue Syndrome (ME/CFS), a debilitating disorder characterized by persistent fatigue and post-exertional malaise (PEM). Particularly in relation to orthostatic intolerance (OI), including postural orthostatic tachycardia syndrome (POTS), this study aimed to investigate the prevalence and clinical relevance of MCA in an Austrian ME/CFS patient cohort. **Methods:** Two data sets were analyzed. The CCCFS data set, a comprehensive, patient-centered online questionnaire consisting of 687 filled surveys, focuses on patient stratification. Self-reported clinical features, disease progression, and treatment responses were analyzed. Preliminary findings were validated in a second, retrospective study, analyzing data of 383 Austrian ME/CFS patients with regard to MCA involvement and OI. **Results:** Among followed-up ME/CFS patients, MCA prevalence increased over the disease course, with up to 25.3% meeting the criteria for clinically relevant MCA. ME/CFS patients with Mast Cell Activation Syndrome (MCAS) and OI reported symptom alleviation significantly more often following mast cell-targeted treatment than those without MCAS (*p* < 0.0001). With regard to IF-channel inhibitors, ME/CFS patients diagnosed with MCAS responded more frequently than those without MCAS (*p* = 0.076), while no significant differences were observed in response to beta blockers (*p* = 0.637). In both cohorts, OI, particularly POTS, was significantly more common in patients with MCA involvement. **Conclusions:** MCA appears to be a frequent and clinically relevant comorbidity in ME/CFS and is associated with a higher prevalence of OI, particularly POTS. Stratifying patients based on MCA involvement may support personalized treatment approaches and improve clinical outcomes.

## 1. Introduction

Approximately 0.3–0.89% of the global population is affected by Myalgic encephalomyelitis/chronic fatigue syndrome (ME/CFS), with a 1.5- to 2-fold higher prevalence in women [1]. ME/CFS is characterized by profound, disabling fatigue and post exertional malaise (PEM), sleep dysfunction and pain, along with neurocognitive, autonomic/orthostatic, neuroendocrine and immunological manifestations [2]. Various disease triggers are described, with viral infections representing the main causes [1]. Detailed knowledge of the mechanisms of disease development and progression remains elusive. Further, the large disease heterogeneity makes it difficult to design and compare studies on ME/CFS. As a result, there is a significant lack of knowledge in key areas such as etiology, diagnostics, and treatment options for the disease [3]. Consequently, patients need to undergo unusually long diagnostic workups to receive an accurate diagnosis [4].

In a subset of ME/CFS patients, clinical features have been observed that suggest increased mast cell activation (MCA). Mast cells are mainly known for their role in atopic diseases and gastrointestinal syndromes, but they also contribute to inflammation via degranulation. Once activated, they release pro-inflammatory mediators. Mechanisms leading to mast cell activation and degranulation are mainly described in response to allergen-stimulated receptor binding. However, various other pathways might contribute to MCA [5,6].

Further, altered cytokine levels that are related to MCA have been observed in ME/CFS [7]. Elevated TNFα levels, in response to MCA, can trigger mast cell activity by further downstream cascade mechanisms and subsequently lead to the release of further inflammatory mediators [8,9]. Also, viral particles may act as antigens, activating immune cells and leading to MCA. Changes in myeloid and lymphocytic immune cells due to these inflammatory and antiviral mechanisms may create an environment resulting in enhancement of MCA [8]. Enhanced MCA may result in excessive mediator release, mitochondrial and vascular damage, as well as hypoperfusion and metabolic disturbances [10]. Furthermore, metabolites, which are known to be altered in post-infectious ME/CFS patients especially under exertion, of the L-arginine and NO pathway, regulate MCA [7,11,12]. And lastly, the known interaction between mast cells and microglial cells in the brain highlights their importance in neuroinflammation [13]. Thus, MCA in ME/CFS could lead to chronic inflammation, contributing to the orthostatic and immune dysregulation that is found in a subgroup of ME/CFS patients [14]. Of interest, the latest insights in MCA revealed a more active mast cell phenotype in female patients [15]. This observation might be associated with the higher prevalence of post-infectious ME/CFS in females.

Based on current knowledge, ME/CFS can only be treated symptomatically. Through adequate disease management and thorough patient stratification, the progression of the illness can be reduced [16,17]. Depending on the symptoms experienced by individual patients, both pharmacological and non-pharmacological treatment options for ME/CFS subgroups are available to alleviate symptoms [17]. To give an example, the pharmacological treatment of ME/CFS patients with orthostatic intolerance (OI) may involve low-dose beta blockers, IF-channel inhibitors, alpha-agonists, mineralocorticoids, or antidiuretics. These medications target the circulatory and autonomic dysfunctions underlying OI, thereby improving patients’ ability to tolerate upright posture and reducing associated fatigue, dizziness, and cognitive impairment. Ultimately, by alleviating OI-specific symptoms, the overall disease burden of ME/CFS is reduced [17,18,19,20,21]. Of interest, an association between neurological or cardiac symptoms, such as cognitive dysfunction or OI, particularly POTS (Postural Orthostatic Tachycardia Syndrome), is described not only in ME/CFS but also in Mast Cell Activation Syndrome (MCAS) patients [22,23,24]. MCAS describes a systemic immune disorder [25,26]. Its symptoms are triggered by recurrent systemic degranulation of mast cells. In contrast to mastocytosis, the cause of symptoms in MCAS is not an increased number of mast cells, but rather a dysfunction and hyperreactivity of these cells, leading to excessive MCA [27]. To what extent MCA may play a role in the pathophysiology of OI in ME/CFS is still not known. By contributing to autonomic nervous system dysfunction, impaired cerebral blood flow, and neuropathic changes, the effects of increased MCA support the notion of shared underlying mechanisms, potentially involving mast cell-mediated pathways.

Our study aims to determine whether a subset of Austrian ME/CFS patients showed increased MCA during their disease and if ME/CFS patients with MCA are more likely to suffer from OI, particularly POTS or orthostatic hypotension (OH). Enhanced MCA has been identified as a contributing factor in the pathophysiology of Post-COVID Syndrome (PCS) [28,29,30]. Based on our findings, we aim to expand knowledge of immunological comorbidities and immunopathogenic components of ME/CFS and to contribute to an improvement in treatment options by stratifying ME/CFS patients based on clinical features.

## 2. Materials and Methods

Motivated by recent literature and our clinical expertise, the aim of a larger project called Computer-based Clustering of Chronic Fatigue Syndrome Patients (CCCFS cohort) was to stratify ME/CFS patients into clinically similar subgroups using a comprehensive online questionnaire to gain pathomechanistic insights into the disease. The online survey was designed in a Patient and Public Involvement and Engagement (PPIE) approach and aims to stratify ME/CFS patients into clinically similar subgroups.

In the study described here, we present a part of the project, which focuses on MCA and orthostatic dysregulation. The patient stratification is based on lifestyle factors, disease progression patterns and medical records from diagnostic workups and treatment approaches. However, due to the format of the online survey, the collected data is based on self-reported answers. Thus, we aimed to confirm the observed findings in subgroups within a second, independent project that aimed to retrospectively analyze an ME/CFS patient cohort regarding MCA and orthostatic dysregulation (ME/CFS-MCA cohort). In both projects only ME/CFS patients over the age of 18 were included. All CCCFS participants gave informed consent before study participation.

### 2.1. CCCFS: Patients

The CCCFS cohort consists of 687 ME/CFS patients from Germany, Austria, Switzerland and parts of Northern Italy between March 2022 and July 2023. To evaluate MCA involvement and orthostatic dysregulation in more detail, patients have been stratified into patients with MCAS diagnosed by a physician, and those without an MCAS diagnosis. Further, a pathological result from a tilt table test or a Schellong/NASA 10 min lean test, as well as the responses to mast cell targeted therapy, beta blockers and IF-channel inhibitors were evaluated. The demographic description of the study CCCFS cohort is listed in Table 1. More detailed information on demographics and disease progression of the cohort has already been discussed elsewhere [4].

### 2.2. ME/CFS-MCA Patients

A retrospective data analysis was performed to further stratify an Austrian ME/CFS patient cohort with regard to MCA and associated OI. The study cohort consists of 383 patients who visited the office of a neurologist in Vienna specializing in ME/CFS between March 2019 and April 2022 and who met the IOM diagnostic criteria for ME/CFS [31]. The diagnosis was made exclusively by the neurologist specialized in ME/CFS.

To evaluate MCA based on the AAAAI criteria [26], patient records were analyzed for the presence of recurrent symptoms of MCA in the following four organ systems: (1) cardiovascular system, with hypotension, tachycardia, syncope, or presyncope; (2) skin, with urticaria, pruritus, flushing, or angioedema; (3) respiratory system, with wheezing, dyspnea, or inspiratory stridor; (4) GI tract, with cramping, pain, diarrhea, nausea, or vomiting. Additionally, the response to mast cell targeted therapy and the presence of elevated concentrations of mast cell mediators or their metabolites were evaluated.

To assess orthostatic dysfunction in the ME/CFS patient cohort, the occurrence of OI, as well as the subtypes POTS and OH, was analyzed. For the diagnoses of “OI”, “POTS”, and “OH”, a pathological result from a tilt table test or a Schellong/NASA 10 min lean test was required. Demographic descriptions of the ME/CFS-MCA cohort, as well as analyzed subgroups, are listed in Table 2.

### 2.3. Data Analysis and Statistics

The data collected was analyzed using descriptive statistics. Absolute and relative frequencies were calculated. To compare the prevalence of OI in ME/CFS patients with and without MCAS diagnosis, as well as positive responses towards mast cell targeted treatment, beta blockers and IF-channel inhibitors in the CCCFS cohort subgroups ME/CFS patients with MCAS and OI and those without MCAS, but OI, fisher’s exact tests were performed. Comparing the prevalence of orthostatic dysregulation, POTS, and orthostatic hypotension between the two subgroups (ME/CFS patients with increased MCA and those without MCA involvement) of the ME/CFS-MCA cohort, fisher’s exact tests were performed as well. Results were considered statistically significant if *p* ≤ 0.05. All descriptive analyses were conducted using GraphPad Prism 9.5.1 (GraphPad Software, Inc., San Diego, CA, USA).

## 3. Results

### 3.1. Evaluation of MCAS and OI in the CCCFS Patient Cohort

By dividing the ME/CFS patients based on diagnosed MCAS into two subgroups, those with MCAS (*n* = 115) and those without MCAS (*n* = 572), we were able to show that 16.7% of the study participants were affected from pathologically enhanced MCA. Only a small proportion of ME/CFS patients (2.8% of the total CCCFS cohort) had already been diagnosed with MCAS prior to their ME/CFS onset.

**Figure 1:**
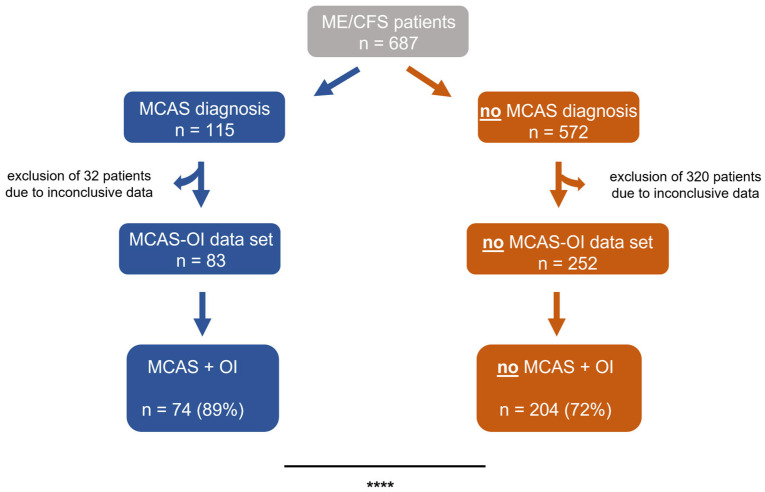
ME/CFS patients were stratified by MCAS diagnosis and OI. Before the final analyses, the data sets of the study population’s subgroups were screened for conclusiveness. Patients with inconclusive results were excluded from the respective subgroups. ME/CFS patients with MCAS were significantly more often diagnosed with OI, than those without (*p* < 0.0001). The prevalence was compared using fisher’s exact tests. **** *p* < 0.0001.

To further stratify the patients and evaluate suspected OI, all ME/CFS patients who did not have a pathological result from a tilt table test or a Schellong/NASA 10 min lean test were excluded from further analyses. Subsequently, patients were divided into an MCAS-OI data set (*n* = 83, 100%) and a no MCAS-OI data set (*n* = 572, 100%). We observed that ME/CFS patients with MCAS were significantly more often diagnosed with OI (89%) compared to ME/CFS patients without MCAS. In this group, only 72% were diagnosed with OI (*p* < 0.0001, Figure 1).

**Figure 2:**
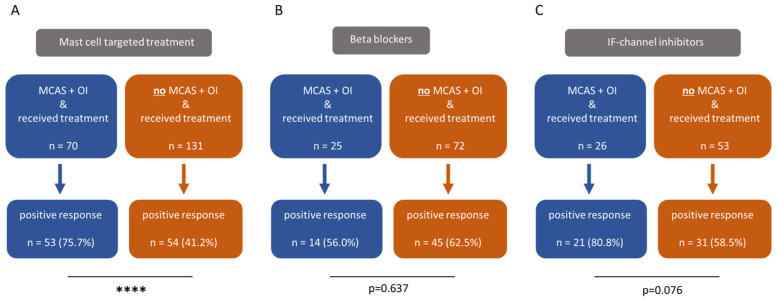
ME/CFS patients with both MCAS and OI respond significantly more often to mast cell targeted treatments than those with OI alone, without a diagnosis of MCAS ((**A**), *p* < 0.0001). Among ME/CFS patients with both MCAS and OI, the response to beta blockers was less frequent compared to those without MCAS (**B**), although this difference was not statistically significant (*p* = 0.637). With regard to IF-channel inhibitors, ME/CFS patients diagnosed with MCAS reported a more frequent positive response than those without MCAS (*p* = 0.076). However, this difference also did not reach statistical significance (**C**). The prevalence was compared using fisher’s exact tests. **** *p* < 0.0001.

Subsequently, both study groups were evaluated for positive responses to mast cell targeted treatment, beta blockers and IF-channel inhibitors (Figure 2). A positive response was defined as “medication leads to a temporary or permanent improvement”. We observed that MCAS-diagnosed ME/CFS patients responded to mast cell targeted treatment significantly more often (Figure 2A) compared to those without an MCAS diagnosis. In terms of responses to beta blockers (Figure 2B), we did not find relevant differences. Although ME/CFS patients diagnosed with MCAS more frequently benefited from IF-channel inhibitors compared to ME/CFS patients without an MCAS diagnosis (Figure 2C), the difference was not statistically significant.

### 3.2. Evaluation of MCA Involvement and OI in the ME/CFS-MCA Patient Cohort

**Figure 3:**
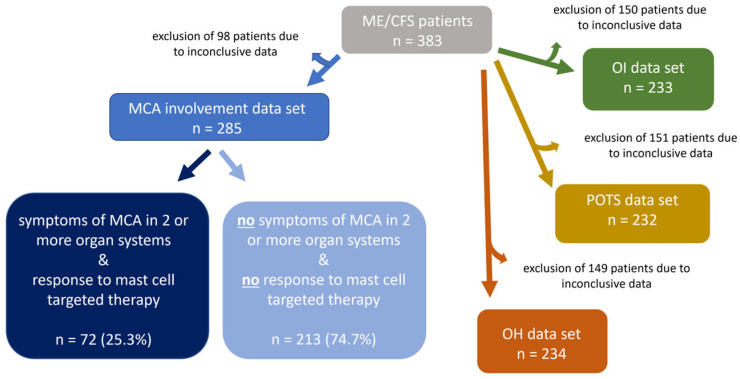
ME/CFS patients were stratified with regard to involvement of MCA and OI. Before the final analyses, the data sets of the study population’s subgroups were screened for conclusiveness. Patients with inconclusive results were excluded from the respective subgroups.

To examine the pathophysiology of increased MCA in ME/CFS and effects of mast cell targeted medication on symptom alleviation, ME/CFS patients were stratified into two subgroups, ME/CFS patients who meet both criteria of “symptoms of MCA in two (or more) organ systems” and “response to mast cell-targeted medication,” and ME/CFS patients who did not meet these criteria. After excluding the 98 patients with inconclusive data sets, the study population was reduced to 285 patients (=100%). Among them, 25.3% (*n* = 72) fulfilled both criteria, “symptoms of MCA in two or more organ systems” and “response to MC medication”, while 74.7% (*n* = 213) did not fulfill these criteria (Figure 3). The third MCAS diagnostic criterion, defined by the AAAAI, elevated concentrations of mast cell mediators or their metabolites, was also recorded for the study patients. However, no statistical analysis could be performed for this parameter, as laboratory testing was conducted in only 3.1% of the ME/CFS patients (*n* = 12). This was considered too small a sample to yield a representative result for the ME/CFS patient cohort under investigation. Moreover, the presence of OI and two subtypes, POTS and OH, was analyzed. Due to inconclusive data in all 3 subgroups, the study population had to be reduced, respectively, to OI *n* = 233, POTS *n* = 232 and OH *n* = 234.

**Figure 4:**
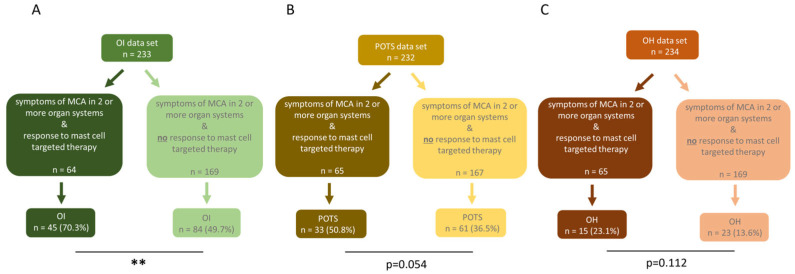
ME/CFS patients with MCA significantly more often experience orthostatic dysregulation than ME/CFS patients without MCA involvement. In ME/CFS patients with MCA, (**A**) OI (*p* = 0.005) was significantly more common compared to those without MCA. (**B**) POTS (*p* = 0.054) and (**C**) OH (*p* = 0.112) were more frequent in the MCA group, but the difference was not statistically significant. The prevalence was compared using fisher’s exact tests. ** *p* < 0.01.

To evaluate the prevalence of OI, ME/CFS patients with symptoms of MCA in two or more organ systems were further divided into two subgroups, ME/CFS patients who responded to mast cell targeted medication and ME/CFS patients who did not respond (Figure 4). In the ME/CFS patient group with increased MCA, OI was significantly more prevalent in 70.3% of patients (*n* = 45), compared to 49.7% (*n* = 84) in the group without MCA (*p* = 0.005; Figure 4A). For the subtype POTS, the prevalence in the group with increased MCA was 50.8% (*n* = 33), compared to 36.5% (*n* = 61) in the group without MCA, thus showing a higher rate in those with MCA. With the higher occurrence of POTS in the MCA group, a statistical trend was observed (*p* = 0.054, Figure 4B). The subtype OH was found in 13.6% (*n* = 23) of patients without MCA and in 23.1% (*n* = 15) of patients with MCA (Figure 4C). The higher occurrence of OH in the MCA group did not reach statistical significance (*p* = 0.112).

## 4. Discussion

According to our CCCFS survey, patients in the German-speaking regions in Germany, Austria, Switzerland, and parts of Northern Italy had been suffering from ME/CFS disease for an average of eight years, while on average receiving a diagnosis only three years prior [4]. This results in an average diagnostic delay of five years before disease-specific treatment, highlighting the urgent need for more thorough patient stratification for specific therapies and improved diagnostic approaches in ME/CFS. To improve current diagnostic standards and tailor symptom-alleviating treatments, we demonstrate the clinical and scientific relevance of MCA and OI in ME/CFS.

### 4.1. A Large Subgroup of ME/CFS Patients Experience MCA Involvement and Respond to Mast Cell Targeted Treatment

The evaluation of the CCCFS cohort showed that an increasing number of ME/CFS patients suffer from MCA with the progression of ME/CFS. While only 2.8% of ME/CFS patients were diagnosed with MCAS before the onset of ME/CFS, 16.7% reported being diagnosed afterwards. This rise in prevalence suggests an increasing involvement of mast cell-associated mechanisms, although a minor bias may arise from improved MCAS recognition by clinicians knowledgeable in immunological mechanisms. Notably, in the CCCFS cohort, ME/CFS patients may still suffer from enhanced MCA, without a formal diagnosis of MCAS, as the diagnostic process remains challenging to this day [25]. Data from the ME/CFS patient cohort revealed that only a small fraction of ME/CFS patients suspected to have MCA involvement had undergone laboratory testing for mast cell mediators or their metabolites. This clearly indicates the necessity of considering laboratory testing for all ME/CFS patients with suspected MCA or a potential diagnosis of MCAS, to identify MCAS in accordance with AAAAI diagnostic criteria. Therefore, we aimed to further elucidate the involvement of MCA in ME/CFS. After careful stratification of the ME/CFS-MCA patient cohort, we observed that 25.3% of ME/CFS patients (*n* = 72) were found to exhibit both symptoms of mast cell activation in two or more of the defined organ systems and a positive response to mast cell-targeted medication. It can be assumed that increased MCA occurs throughout the course of disease in a substantial proportion of ME/CFS patients. Moreover, we observed symptom improvement in the subgroup of ME/CFS patients showing symptoms of MCA after receiving mast cell targeted therapy. This highlights the clinical relevance of MCA-oriented patient stratification and mast cell-targeted therapies. Not only can symptom alleviation significantly improve patients’ quality of life, but such therapies are also generally considered safe when appropriately prescribed and monitored. Many patients respond well to mediator-blocking drugs such as antihistamines or mast cell stabilizers, which have favorable safety profiles. However, newer and more aggressive therapies, especially those targeting mast cell proliferation, require careful monitoring for potential side effects [32]. Lastly, as ME/CFS and PCS of the ME/CFS type represent closely related disease entities, pathogenic mechanisms may overlap between the two. Therefore, systematically assessing symptoms indicative of MCA is not only important for clinical practice, but it is also of special relevance for elucidating ME/CFS pathology in future scientific studies.

### 4.2. ME/CFS Patients with MCA Involvement Experience OI Significantly More Often, Particularly Its Subtype POTS

Recent literature reports a higher prevalence of OI, particularly POTS, in patients with MCAS [22,23,24]. To investigate whether this relationship also exists in ME/CFS patients and whether the prevalence of OI differs in association with MCA, the ME/CFS cohorts studied were analyzed for OI-related diagnoses and responses to medication. In the CCCFS cohort, we observed a significantly higher prevalence of OI in ME/CFS with MCAS compared to ME/CFS patients without MCAS. Moreover, with regard to symptom alleviation, we observed that patients with MCAS and OI benefited from mast cell targeted treatment significantly more compared to those without MCAS. Since MCAS-OI patients often exhibit a characteristic hyperadrenergic response, marked by excessive activation of the sympathetic nervous system, which can be triggered by mast cell mediators, mast cell targeted medication may represent a safer alternative to beta blockers in treatment approaches [33]. Moreover, this subgroup did not report greater symptom improvement after receiving beta blockers compared to ME/CFS patients without MCAS. Interestingly, ME/CFS patients with both MCAS and OI more frequently, though not significantly, reported symptom improvement following treatment with IF-channel inhibitors. IF-channel inhibitors, commonly used in angina pectoris and heart failure, are increasingly prescribed off-label for POTS, particularly the hyperadrenergic POTS subtype, where patients experience excessive tachycardia upon standing [34,35]. Given that many MCAS patients exhibit autonomic nervous system dysfunction, including hyperadrenergic symptoms, the therapeutic effect is plausible. Another explanation might be that beta blockers reduce blood pressure, which may be disadvantageous in the context of histamine-induced vasodilation, whereas IF-channel inhibitors do not exert this effect. However, to the best of our knowledge, there is no direct mechanistic link between IF-channel inhibitors and MCAS in terms of mast cell biology. To elucidate the connection between OI and MCAS and further evaluate the pathological mechanisms of ME/CFS, future studies should emphasize the different effects of already used medication. The mode of action of different classes of medication may offer insights into the underlying pathways involved in the pathogenesis of ME/CFS. To further investigate this observation, we analyzed the ME/CFS-MCA cohort for OI, with a focus on the subtypes POTS and OH. The prevalence rates in the subgroup of ME/CFS patients with increased MCA were compared with those in ME/CFS patients without MCA. In the ME/CFS patient group with increased MCA, patients more often experienced OI and POTS, compared to the group without MCA. OH was observed more frequently, however the difference did not reach statistical significance. Future research should emphasize distinct mechanistic pathways, considering the growing evidence on non-IgE mediated mechanisms of MCA via alternative receptors [36,37,38]. Notably, the receptor MRGPRX2 has been shown to be activated by various small agents leading to enhanced MCA and contributing to neuroinflammation. Additionally, analyses of mitochondrial respiratory complex integrity and membrane potential have showed critical associations with mast cell degranulation.

### 4.3. Study Limitations

Limitations of our study include its reliance on self-reported data in the CCCFS patient cohort, the retrospective design of the ME/CFS-MCA patient cohort, and potential differences in MCAS diagnostic procedure. The primary objective of the here described studies was to identify clinically meaningful subgroups of ME/CFS patients based on self-reported symptoms, lifestyle factors, disease characteristics and already existing medical reports, rather than to perform biological validation. This design enables the identification of patterns and hypotheses that can later be tested in smaller, clinically verified cohorts under controlled laboratory conditions. Furthermore, results from Austrian cohorts may not be fully generalizable to other populations due to national differences in health care systems.

## 5. Conclusions

Overall, our findings suggest that the prevalence of MCA increases with ME/CFS disease progression. When accurately diagnosed, approximately 25% of ME/CFS patients present with involvement of MCA as a comorbidity. A substantial proportion of these patients benefit from mast cell targeted therapies, which not only lead to symptom improvement but also reduce overall disease burden. Additionally, we observed that the prevalence of OI, particularly POTS, is higher among ME/CFS patients with increased MCA than in those without. The insights carry important clinical implications, as both patients and health care providers may benefit from increased awareness of the link between MCA and OI, especially POTS. In ME/CFS, patient stratification is essential for tailoring treatment approaches to individual needs and to ultimately improve outcomes. Systematic assessment of symptoms suggestive of MCA is not only critical in clinical practice. Examining overlapping pathogenic mechanisms is also highly relevant for advancing our understanding of ME/CFS pathophysiology through the exploration of overlapping mechanisms in future research. Laboratory testing would undoubtedly be highly valuable for validating our findings and for further deepening the understanding of the underlying pathophysiological mechanisms. In the context of ME/CFS, clinicians need to emphasize more on patient stratification and the identification of clinically relevant subgroups. To support this approach, broader dissemination of knowledge about the disease among medical professionals is essential.

## Figures and Tables

**Table 1:** Demographic description of the CCCFS patient cohort. The data point “sex” at the time point of study inclusion was defined as female, male or diverse, in case participants underwent hormonal therapy for gender affirmation.

ME/CFS Data Sets	ME/CFS (Total)	MCAS Diagnosis	No MCAS Diagnosis
Participants	687 (100%)	115 (16.7%)	572 (83.3%)
Sex (f/m/d)	84%/15.6%/0.4%	87.0%/11.3%/1.7%	83.4%/16.4%/0.2%
Age, median (IQR)	41 (33.0–50.0)	39 (32.0–48.5)	41 (33.0–50.0)

**Table 2:** Demographic description of the ME/CFS-MCA patient cohort. The stated percentage of the number of participants refers to the number of ME/CFS participants included in the study with complete and conclusive data sets. Patients with incomplete or inconclusive results were excluded from the respective subgroups.

ME/CFS Data Sets	ME/CFS (Total)	MCA	OI	POTS	OH
Participants	383 (100%)	285 (74%)	233 (61%)	232 (61%)	234 (61%)
Sex, female (%)	257 (67%)	192 (67%)	154 (66%)	153 (66%)	156 (67%)
Age, median (IQR)	42 (33.0–52.5)	41 (33.0–52.0)	42 (33.0–52.0)	41 (33.0–52.0)	41 (33.0–52.0)

## Data Availability

The raw data supporting the conclusions of this article will be made available by the authors on request.

## Abbreviations

The following abbreviations are used in this manuscript:

AAAAI	American Academy of Allergy, Asthma & Immunology
CCCFS	Computer-based Analysis of Chronic Fatigue Syndrome Patients
MCA	Mast Cell Activation
MCAS	Mast Cell Activation Syndrome
ME/CFS	Myalgic Encephalomyelitis / Chronic Fatigue Syndrome
OH	Orthostatic Hypotension
OI	Orthostatic intolerance
PCS	Post-COVID Syndrome
PEM	Post-exertional Malaise
POTS	Postural Tachycardia Syndrome
PPIE	Patient and Public Involvement and Engagement

## References

[1] Lim E.J., Ahn Y.C., Jang E.S., Lee S.W., Lee S.H., Son C.G. (2020). Systematic review and meta-analysis of the prevalence of chronic fatigue syndrome/myalgic encephalomyelitis (CFS/ME). J. Transl. Med..

[2] Carruthers B.M., van de Sande M.I., De Meirleir K.L., Klimas N.G., Broderick G., Mitchell T., Staines D., Powles A.C.P., Speight N., Vallings R. (2011). Myalgic encephalomyelitis: International Consensus Criteria. J. Intern. Med..

[3] Bested A.C., Marshall L.M. (2015). Review of Myalgic Encephalomyelitis/Chronic Fatigue Syndrome: An evidence-based approach to diagnosis and management by clinicians. Rev. Env. Health.

[4] Hainzl A., Rohrhofer J., Schweighardt J., Hermisson J., Hoffmann K., Komenda-Lett M., Schlaff G., Schulz C., Stingl M., Thonhofer K. (2024). Care for ME/CFS—Praxisleitfaden für die Versorgung von ME/CFS Betroffenen. Zenodo. https://share.google/q5AR8MToEKdKCr99G.

[5] Kim D.Y., Lee J.S., Park S.Y., Kim S.J., Son C.G. (2020). Systematic review of randomized controlled trials for chronic fatigue syndrome/myalgic encephalomyelitis (CFS/ME). J. Transl. Med..

[6] Agier J., Pastwińska J., Brzezińska-Błaszczyk E. (2018). An overview of mast cell pattern recognition receptors. Inflamm. Res..

[7] Glass K.A., Germain A., Huang Y.V., Hanson M.R. (2023). Urine Metabolomics Exposes Anomalous Recovery after Maximal Exertion in Female ME/CFS Patients. Int. J. Mol. Sci..

[8] Gu Y., Yang D.K., Spinas E., Kritas S.K., Saggini A., Caraffa A., Antinolfi P., Saggini R., Conti P. (2015). Role of TNF in mast cell neuroinflammation and pain. J. Biol. Regul. Homeost. Agents.

[9] Rijnierse A., Koster A.S., Nijkamp F.P., Kraneveld A.D. (2006). TNF-alpha is crucial for the development of mast cell-dependent colitis in mice. Am. J. Physiol. Gastrointest. Liver Physiol..

[10] Zhang B., Asadi S., Weng Z., Sismanopoulos N., Theoharides T.C. (2012). Stimulated human mast cells secrete mitochondrial components that have autocrine and paracrine inflammatory actions. PLoS ONE.

[11] Giraldelo C.M., Zappellini A., Muscará M.N., De Luca I.M., Hyslop S., Cirino G., Zatz R., De Nucci G., Antunes E. (1994). Effect of arginine analogues on rat hind paw oedema and mast cell activation in vitro. Eur. J. Pharmacol..

[12] Coleman J.W. (2002). Nitric oxide: A regulator of mast cell activation and mast cell-mediated inflammation. Clin. Exp. Immunol..

[13] Dileepan K.N., Raveendran V.V., Sharma R., Abraham H., Barua R., Singh V., Sharma R., Sharma M. (2023). Mast cell-mediated immune regulation in health and disease. Front. Med..

[14] Wirth K.J., Scheibenbogen C. (2021). Pathophysiology of skeletal muscle disturbances in Myalgic Encephalomyelitis/Chronic Fatigue Syndrome (ME/CFS). J. Transl. Med..

[15] Mackey E., Thelen K.M., Bali V., Fardisi M., Trowbridge M., Jordan C.L., Moeser A.J. (2020). Perinatal androgens organize sex differences in mast cells and attenuate anaphylaxis severity into adulthood. Proc. Natl. Acad. Sci. USA.

[16] Rowe P.C., Underhill R.A., Friedman K.J., Gurwitt A., Medow M.S., Schwartz M.S., Speight N., Stewart J.M., Vallings R., Rowe K.S. (2017). Myalgic Encephalomyelitis/Chronic Fatigue Syndrome Diagnosis and Management in Young People: A Primer. Front. Pediatr..

[17] Bateman L., Bested A.C., Bonilla H.F., Chheda B.V., Chu L., Curtin J.M., Dempsey T.T., Dimmock M.E., Dowell T.G., Felsenstein D. (2021). Myalgic Encephalomyelitis/Chronic Fatigue Syndrome: Essentials of Diagnosis and Management. Mayo Clin. Proc..

[18] Collatz A., Johnston S.C., Staines D.R., Marshall-Gradisnik S.M. (2016). A Systematic Review of Drug Therapies for Chronic Fatigue Syndrome/Myalgic Encephalomyelitis. Clin. Ther..

[19] Williams E.L., Raj S.R., Schondorf R., Shen W.K., Wieling W., Claydon V.E. (2022). Salt supplementation in the management of orthostatic intolerance: Vasovagal syncope and postural orthostatic tachycardia syndrome. Auton. Neurosci..

[20] Snapper H., Cheshire W.P. (2022). Oral and intravenous hydration in the treatment of orthostatic hypotension and postural tachycardia syndrome. Auton. Neurosci..

[21] Miller A.J., Raj S.R. (2018). Pharmacotherapy for postural tachycardia syndrome. Auton. Neurosci..

[22] Wang E., Ganti T., Vaou E., Hohler A. (2021). The relationship between mast cell activation syndrome, postural tachycardia syndrome, and Ehlers-Danlos syndrome. Allergy Asthma Proc..

[23] Novak P., Giannetti M.P., Weller E., Hamilton M.J., Castells M. (2022). Mast cell disorders are associated with decreased cerebral blood flow and small fiber neuropathy. Ann. Allergy Asthma Immunol..

[24] Kohno R., Cannom D.S., Olshansky B., Xi S.C., Krishnappa D., Adkisson W.O., Norby F.L., Fedorowski A., Benditt D.G. (2021). Mast Cell Activation Disorder and Postural Orthostatic Tachycardia Syndrome: A Clinical Association. J. Am. Heart Assoc..

[25] Weiler C.R., Austen K.F., Akin C., Barkoff M.S., Bernstein J.A., Bonadonna P., Butterfield J.H., Carter M., Fox C.C., Maitland A. (2019). AAAAI Mast Cell Disorders Committee Work Group Report: Mast cell activation syndrome (MCAS) diagnosis and management. J. Allergy Clin. Immunol..

[26] Valent P., Akin C., Bonadonna P., Hartmann K., Brockow K., Niedoszytko M., Nedoszytko B., Siebenhaar F., Sperr W.R., Oude Elberink J.N.G. (2019). Proposed Diagnostic Algorithm for Patients with Suspected Mast Cell Activation Syndrome. J. Allergy Clin. Immunol. Pract..

[27] Akin C., Valent P., Metcalfe D.D. (2010). Mast cell activation syndrome: Proposed diagnostic criteria. J. Allergy Clin. Immunol..

[28] Weinstock L.B., Brook J.B., Walters A.S., Goris A., Afrin L.B., Molderings G.J. (2021). Mast cell activation symptoms are prevalent in Long-COVID. Int. J. Infect. Dis..

[29] Sumantri S., Rengganis I. (2023). Immunological dysfunction and mast cell activation syndrome in long COVID. Asia Pac. Allergy.

[30] Untersmayr E., Venter C., Smith P., Rohrhofer J., Ndwandwe C., Schwarze J., Shannon E., Sokolowska M., Sadlier C., O’Mahony L. (2024). Immune Mechanisms Underpinning Long COVID: Collegium Internationale Allergologicum Update 2024. Int. Arch. Allergy Immunol..

[31] National Academy of Sciences (US) (2015). The National Academies Collection: Reports funded by National Institutes of Health. Beyond Myalgic Encephalomyelitis/Chronic Fatigue Syndrome: Redefining an Illness.

[32] Molderings G.J., Haenisch B., Brettner S., Homann J., Menzen M., Dumoulin F.L., Panse J., Butterfield J., Afrin L.B. (2016). Pharmacological treatment options for mast cell activation disease. Naunyn Schmiedebergs Arch. Pharmacol..

[33] Shibao C., Arzubiaga C., Roberts L.J., Raj S., Black B., Harris P., Biaggioni I. (2005). Hyperadrenergic postural tachycardia syndrome in mast cell activation disorders. Hypertension.

[34] Thollon C., Vilaine J.P. (2010). I(f) inhibition in cardiovascular diseases. Adv. Pharmacol..

[35] Taub P.R., Zadourian A., Lo H.C., Ormiston C.K., Golshan S., Hsu J.C. (2021). Randomized Trial of Ivabradine in Patients with Hyperadrenergic Postural Orthostatic Tachycardia Syndrome. J. Am. Coll. Cardiol..

[36] Ogasawara H., Noguchi M. (2021). Therapeutic Potential of MRGPRX2 Inhibitors on Mast Cells. Cells.

[37] Piotin A., Oulehri W., Charles A.-L., Tacquard C., Collange O., Mertes P.-M., Geny B. (2024). Oxidative Stress and Mitochondria Are Involved in Anaphylaxis and Mast Cell Degranulation: A Systematic Review. Antioxidants.

[38] Wollam J., Solomon M., Villescaz C., Lanier M., Evans S., Bacon C., Freeman D., Vasquez A., Vest A., Napora J. (2024). Inhibition of mast cell degranulation by novel small molecule MRGPRX2 antagonists. J. Allergy Clin. Immunol..

